# Assessment of the performance of Ni carbon nanotube nano composite coatings and activated carbon for diesel exhaust treatment

**DOI:** 10.1038/s41598-025-88823-6

**Published:** 2025-02-06

**Authors:** Gamal E. M. Nasr, Magdy A. Baiomy, A. Z. Taieb, Mayada E. Abdel Razek, Mohamed Refai

**Affiliations:** 1 Agricultural Engineering Department, Fac. Agric, Cairo University, Giza, 12613 Egypt; 2 Bio-Engineering Dept., Agric. Engineering Research Institute, Agric. Research Center, Dokki, Giza, Egypt; 3 Agricultural Engineering Department, Faculty of Agriculture, Agric. Engineering Sector, Cairo University, Ministry of Agric, Dokki, Giza Egypt

**Keywords:** Emission Control, Diesel Engine, Emission control technologies, Diesel treatment device, Material Treatment, Environmental sciences, Materials science

## Abstract

Diesel engines are essential in sectors such as transportation, agriculture, and power generation, offering benefits like fuel efficiency, high power output, and durability. However, their emissions (NO_X_, CO_2_, CO, HC, SO_2_, and PM) significantly contribute to air pollution, posing serious environmental and health risks. This study aimed to design and fabricate a unit that simulates diesel engine emissions and tests various purification materials. The unit consists of a combustion chamber, filtration media, and exhaust pipes, with materials such as activated carbon, activated carbon with magnesium oxide, and Ni-**Carbon Nanotube (**CNTs) nanocomposites tested under controlled combustion conditions to measure their pollutant removal efficiencies. Results showed that 100% activated carbon achieved pollutant removal efficiencies of 85.21% for CO_2_, 80.77% for CO, and 68.84% for HC. Combining activated carbon with magnesium oxide (AC: MgO) enhanced these efficiencies to 76.92% for CO_2_, 86.84% for CO, and 73.28% for HC. Ni-CNTs nanocomposites (at 0.2 concentration) demonstrated the highest performance, with removal efficiencies of 93.13% for CO_2_, 94.87% for CO, and 76.02% for HC. These results emphasize the potential of Ni-CNTs nanocomposites as highly efficient materials for reducing diesel exhaust emissions, contributing significantly to cleaner air, better public health, and more sustainable diesel technologies.

## Introduction

Environmental degradation and declining air quality have reached unprecedented levels due to the extensive dependence on fossil fuels in industry, transportation, agriculture, and construction sectors. These activities are among the leading contributors to air pollution, one of the most critical challenges facing the world today, particularly in low- and middle-income countries. According to the World Health Organization (WHO), more than 90% of the 7 million annual deaths associated with air pollution occur in these regions^1,2^. The burning of fossil fuels is a primary driver of air pollution, leading to the emission of harmful pollutants and exacerbating global warming^3^. This poses a severe threat to humanity, as millions suffer the catastrophic effects of climate change, including extreme weather events. The Intergovernmental Panel on Climate Change (IPCC) has emphasized the urgent need to control rising temperatures and mitigate global warming to prevent irreversible damage. Fossil fuel emissions surged during the 19th century, reaching critical levels, with the energy, transportation, construction, and agriculture sectors being key contributors. Despite the scientific consensus on the causes and effects of global warming, debates over appropriate mitigation strategies continue to persist in both political and public spheres^4^. Diesel engines, valued for their efficiency and low maintenance, significantly contribute to environmental pollution by emitting harmful gases such as PM, CO, CO₂, HC, SO₂, and NOx^5^. These emissions contribute to climate change, impacting both the environment and public health^6^. One of the key trends is the shift to renewable energy sources to reduce emissions and promote economic growth^7^.

The combustion of fossil fuels in diesel engines produces a mixture of toxic pollutants that contribute to the formation of smog, acid rain, and the depletion of atmospheric ozone. Efforts to mitigate these emissions are crucial, yet existing measures often fall short of achieving substantial reductions^8^. In Egypt, air pollution, particularly CO₂ emissions, amounts to 204.3 million tons, ranking the country 29th globally and third in the Arab world^9^. CO a colorless, odorless, and toxic gas, is one of the primary air pollutants produced by the incomplete combustion of highly stable fuels. The permissible limit for CO is set at 30 µg/m² per hour, according to Egyptian Environmental Law. NOx which account for 40–70% of global pollution levels, play a critical role in the formation and transformation of other pollutants, including ozone, particulate matter, and acid rain. Diesel engines contribute approximately 85% of NOx emissions in the atmosphere^10^. The same environmental law establishes the permissible limit for SO₂ emissions at 400 µg/m² per hour. Major sources of SO₂ emissions include fossil fuel combustion in power plants (73%) and other industrial facilities (20%), with coal and oil deposits typically containing 1–2% S.^11^. According to Egyptian regulations, the permissible limits for sulfur dioxide concentrations over a 24-hour period are 125 and 150 µg/m², with a maximum hourly concentration of 350 µg/m², which is more than five times the recommended limits set by the WHO. Additionally, (HC) emissions, which naturally occur at high loads, have significant environmental impacts, primarily contributing to the formation of ground-level ozone. This ozone formation has widespread detrimental effects on both human health and ecosystems^12^.

Advanced filtration technologies have emerged as a promising avenue to combat emissions to address the negative impact of these emissions, various mitigation methods have been employed, including the use of catalytic converters, (DPF), (SCR) systems, (EGR) devices, and advanced filtration technologies that utilize activated carbon and nanoparticles. Activated carbon, known for its high porosity, surface area, and chemical stability, has been widely used for gas adsorption in industrial applications^13^. Additionally, integrating hydrogen with sapota seed biodiesel enhances engine performance, reduces emissions, and provides a viable alternative to diesel in dual-fuel diesel engines^14^. One method to reduce emissions is converting used pork fat oil into biodiesel through transesterification for use in CRDI diesel engines, with Di-tert-butyl peroxide (DTBP) added as an ignition enhancer. A blend of diesel and pork fat biodiesel (B20DTBP10) was tested under various operating conditions, such as compression ratios and injection pressures. The results showed that higher compression ratios and injection pressures improved thermal efficiency, reduced C and HC emissions, but increased (NOx) emissions^15^.

Despite advancements in filtration media, challenges remain in optimizing the performance and cost-effectiveness of these technologies for widespread adoption. Existing research often focuses on laboratory-scale applications, leaving a gap in understanding their real-world performance under varying operational conditions. Recent studies have shown that modifying (AC) with (MgO) enhances its adsorption capacity, leading to a significant improvement in the removal of harmful gases such as CO and HC. Previous studies have tested the performance of AC modified with MgO, demonstrating significant improvements in gas adsorption. Specifically, the enhanced pore structure of activated carbon, achieved through a 70:30 (MgO: AC), resulted in adsorption efficiencies of 99.14% for CO and 87.73% for HC, highlighting its effectiveness in treating exhaust gases^16^. AC is widely used in industrial applications as an adsorbent due to its high porosity, large surface area, and diverse surface chemical properties. These characteristics, along with its affordability and chemical stability, make AC a vital component in numerous fields^17^. Commercial activated charcoal typically has an adsorption efficiency of about 70%. However, activated carbon derived from coconut shells shows a remarkable NO elimination efficiency, exceeding 86.4% ^18^. Nanomaterials, such as calcium oxide (CaO), aluminum oxide (Al₂O₃), (MgO), and (CNTs), have demonstrated high efficiency in adsorbing exhaust gases. The addition of aluminum oxide nanoparticles improves engine performance and reduces emissions^19^. Alternative fuels and nanomaterials enhance engine efficiency and reduce emissions, supporting the transition to sustainable diesel technologies and showcasing innovation in energy solutions^20^. Among these, carbon nanotubes (CNTs) stand out due to their exceptional adsorption properties, which stem from their large surface area and unique structural characteristics^21,22^ to achieve exceptional adsorption efficiency, exceeding 99% ^23^. These features make CNTs particularly suitable for use in nanocomposite coatings designed for gas treatment. While significant advancements have been made in laboratory-scale studies of activated carbon and nanomaterials for emission control, their practical applications under real-world conditions remain underexplored. Additionally, challenges related to cost-effectiveness, compliance with environmental standards, and region-specific studies, particularly in countries like Egypt, highlight the need for further research.

This research aims to design and evaluate a model that simulates diesel engine emissions, focusing on assessing the efficiency of various filtration media for gas treatment. It seeks to understand the composition of emissions, develop mathematical and physical models, and identify the most effective media for adsorbing gases produced by fossil fuel combustion. The study also emphasizes the enhancement of treatment technologies, the reduction of environmental impacts, and ensuring compliance with environmental standards. Ultimately, the research aims to improve air quality, minimize pollution, and protect both the environment and human health. The novelty of this research lies in its focus on testing new filtration materials for purifying gases resulting from fossil fuel combustion. This approach addresses challenges such as cost-effectiveness and environmental compliance, particularly in developing nations like Egypt. By applying advanced filtration technologies under real-world conditions, the study fills a gap in prior research and contributes to scalable, innovative solutions for emission control.

## Materials and methods

To achieve the objective of this study, (AC), (AC-MgO), and Ni-CNT_S_ nanocomposite coating were selected for evaluation. The Ni-CNT_S_ nanocomposite was deposited onto a stainless steel screen with 100 holes per mesh. All materials were tested using a specially designed device that includes fossil coal, diesel fuel, an oxygen source, and the materials under evaluation.

**Fig. 1:**
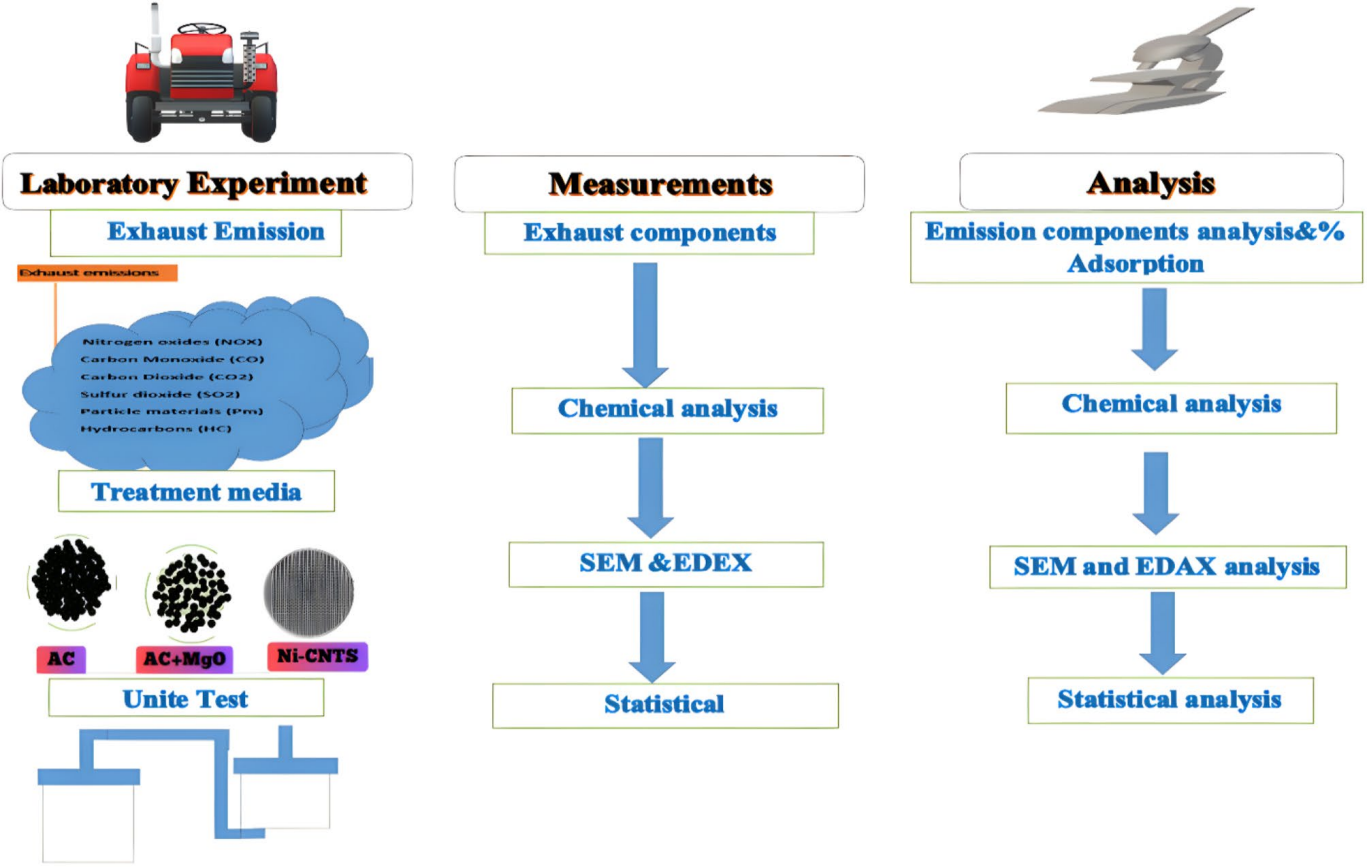
Shows the setup of the experiment.

The process involves constructing a refractory combustion chamber where fossil coal is burned with diesel fuel. Oxygen is supplied through an opening at the bottom to sustain combustion and gas emissions. The emitted gases are directed through outlet pipes to a filtration chamber containing the evaluation materials, through which the gases pass. Pollutant concentrations are measured to assess the effectiveness of the different treatment materials. Figure 1 shows the experimental setup. The illustration was created using Adobe Photoshop 2023.

### Characterization and performance evaluation of the filter media

#### Properties of activated carbon

AC is widely used in industrial applications as an adsorbent and a key component in many processes due to its high porosity, large surface area, and diverse surface chemical properties^24^. Additionally, it is cost-effective, chemically stable, and exhibits excellent adsorption capabilities. Coconut shell-derived activated carbon, in particular, is known for its high adsorption efficiency, with a removal efficiency of over 84.4% ^25^.

AC is also a popular adsorbent for SO_2_ due to its significant porosity, excellent thermal stability, and the presence of oxygen-containing functional groups on its surface. These features enhance its ability to adsorb contaminants and provide more active sites for the removal of sulfur dioxide and other pollutants^24^. Table 1 shows the Chemical analysis of activated carbon.

**Table 1:** Chemical analysis of activated carbon.

Moisture %	Pb (mg/kg)	As (mg/kg)	Cd (mg/kg)	Hg (mg/kg)	Total Ash%	PH	C%	*N*%	S%
3.2	1.565	0.387	0.543	0.324	0.654	7.56	83.56	1.33	1.53

The specific density of activated carbon was measured, with an average value of 1 g/cm³. The diameters of the activated carbon particles were measured, ranging from 0.6 to 1 mm.”

#### Enhancement of adsorption capacity in activated carbon with MgO

MgO was used to enhance the quality and adsorption capacity of AC derived from durian peel. Impregnating AC with MgO is a common method for improving its adsorption properties, as MgO increases surface area and boosts the carbon’s adsorption capacity. MgO particles are particularly effective in adsorbing carbon dioxide, as they are chemically produced and combined with activated carbon to improve adsorption efficiency. Metals like MgO are used to modify AC for enhanced adsorption, with studies showing that MgO can achieve a surface area of up to 400 m²/g^26^.

AC derived from coconut shells was modified by incorporating MgO in varying ratios to enhance its adsorption capacity and efficiency in filtering exhaust gases. Three AC: MgO configurations by weight were evaluated:

- 7:0.5 ratio, consisting of 331 g of AC and 24 g of MgO.
- 7:1 ratio, consisting of 310 g of AC and 45 g of MgO.
- 7:1.5 ratio, consisting of 292 g of AC and 63 g of MgO.

The filter discs were manufactured with precise dimensions to ensure consistency during testing. The MgO used in the modification had a high purity of 97%, which significantly contributed to the performance of the enhanced AC.

#### Electroless deposition of Ni-CNTs onto stainless steel mesh

**Fig. 2:**
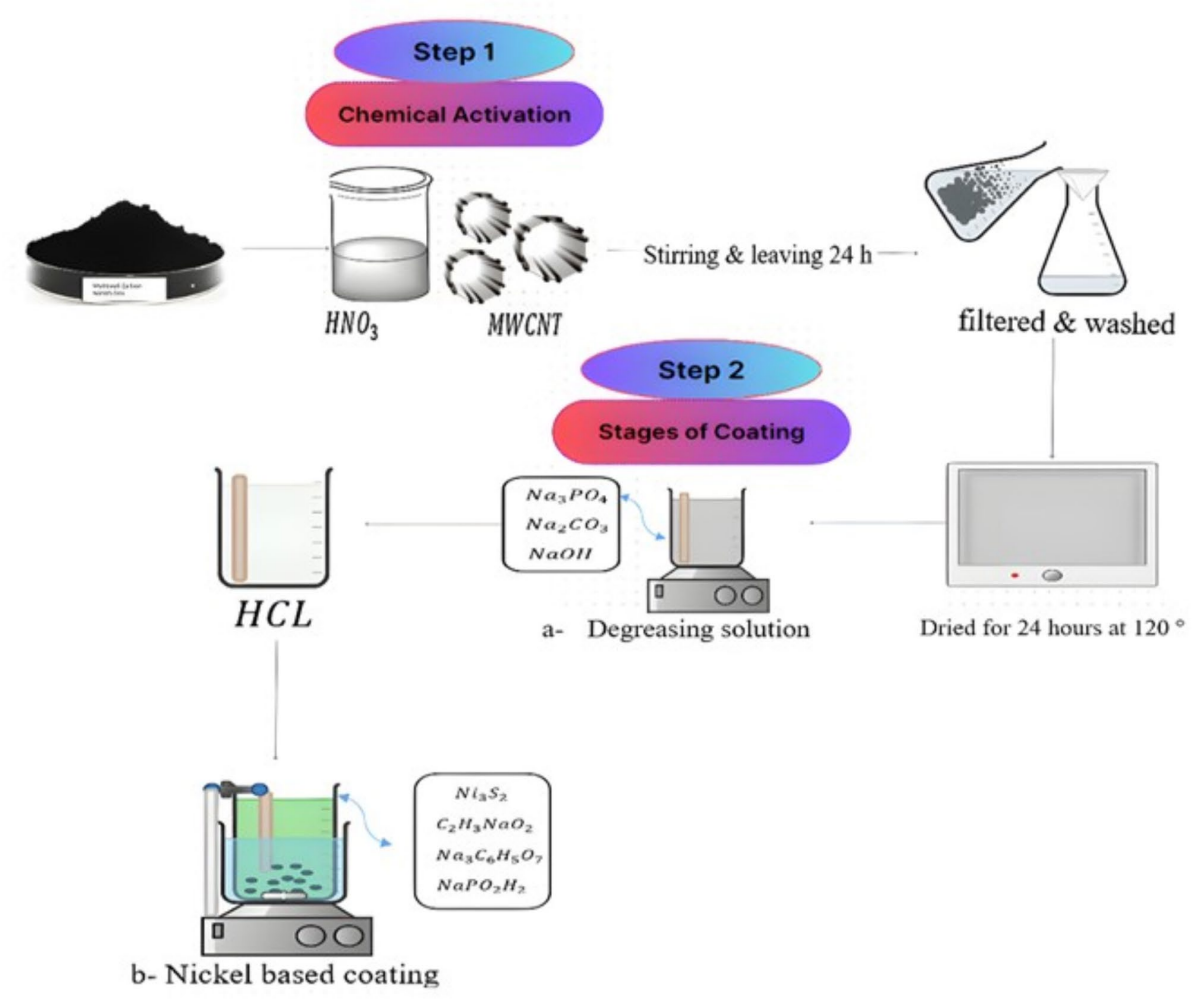
The steps and techniques of Ni-CNT Nanocomposite Coating on stainless steel sheet.

The electroless deposition technique was employed to coat stainless steel woven mesh screens with Ni-CNTs layers. The chemical composition of the stainless steel mesh was analyzed using an optical emission spectrometer (DV6–BAIRD), which confirmed the material’s standard properties. The analysis revealed the following composition: 0.67 wt% Fe, 0.2 wt% Mn, 0.1 wt% Si, 0.045 wt% P, and 0.03 wt% S. The mesh samples had a diameter of 150 mm, with wire diameters ranging from 0.02 mm (0.0008”) to 2.0 mm (0.08”), and a mesh count of 100 openings per inch. These specifications ensure uniformity, structural stability, and suitability for applications requiring enhanced coating adhesion and efficient filtration Fig. 2 illustrates the electroless coating process. The procedure begins with the activation of multi-walled carbon nanotubes (CNTs) to enhance their surface characteristics. First, a nitric acid solution is prepared in a 1:4 ratio (nitric acid to water). To this solution, 0.5 g of CNTs are added and stirred for 30 min before being left to rest for 24 h to allow the acid to react effectively^21^. Afterward, the mixture is filtered using ultra-fine filter paper, and the CNTs are washed with distilled water until the pH reaches 7. The activated CNTs are then dried at 120 °C for 24 h, making them ready for subsequent use in the coating process.

Next, the stainless steel substrate is prepared by removing oils and grease from its surface. This is achieved by immersing the substrate in an alkaline degreasing solution consisting of 20 g/L of trisodium phosphate (Na₃PO₄), sodium carbonate (Na₂CO₃), and sodium hydroxide (NaOH), maintaining the mixture at 70 °C for 3 min. After this, the oxide layer on the substrate is removed by immersing it in a 10% hydrochloric acid (HCl) solution for one minute at room temperature. The substrate is then rinsed with distilled water to remove any residual acid, making it ready for the coating solution.

The coating solution is prepared by dissolving nickel sulfate (35 g/L), sodium acetate (15 g/L), trisodium citrate (5 g/L), and sodium hypophosphite (10 g/L) in one liter of deionized water. To optimize the distribution of CNTs within the solution and prevent their agglomeration, the electrolyte is agitated using a magnetic stirrer at 400 rpm for at least two hours, followed by sonication for 30 min. During the deposition process, the stirring rate is maintained at 250 rpm to ensure uniform dispersion and effective coating application. Additionally, five different concentrations of Ni-CNTs nanocomposite coatings are tested: 0.1, 0.2, 0.3, 0.4, and 0.5 g/L, enabling the evaluation of various loading levels for optimal performance.

This process ensures both the proper activation of CNTs and the thorough preparation of the substrate, contributing to the successful application of the electroless coating.

### Design of the exhaust gas filtration test unit

The test unit is designed to assess the performance of various materials for exhaust gas filtration. It is consists of the following key components:

- **Cylindrical Combustion Chamber**: This chamber is equipped with a circular perforated strainer, on which the material is placed, allowing for the generation of exhaust gases from sources such as charcoal and diesel fuel.
- **Combustion Chamber Cover**: The cover is centrally perforated and is connected to the filtration chamber via a 20 mm diameter pipe.
- **Filtration Chamber**: A cylindrical chamber that houses the filtration media for exhaust gas treatment.
- **Connecting Pipe**: A 20 mm diameter pipe runs from the combustion chamber cover to the bottom of the filtration chamber, facilitating the transfer of exhaust gases.
- **Filtration Media Chamber**: This chamber features a cylindrical bottom made of stainless steel wire, providing easy access to the treated emissions.

#### Exhaust Gas Discharge

The chamber is equipped with a cylindrical cover featuring a central hole, along with a 20 mm diameter pipe welded to it for discharging the exhaust gases.

**Fig. 3:**
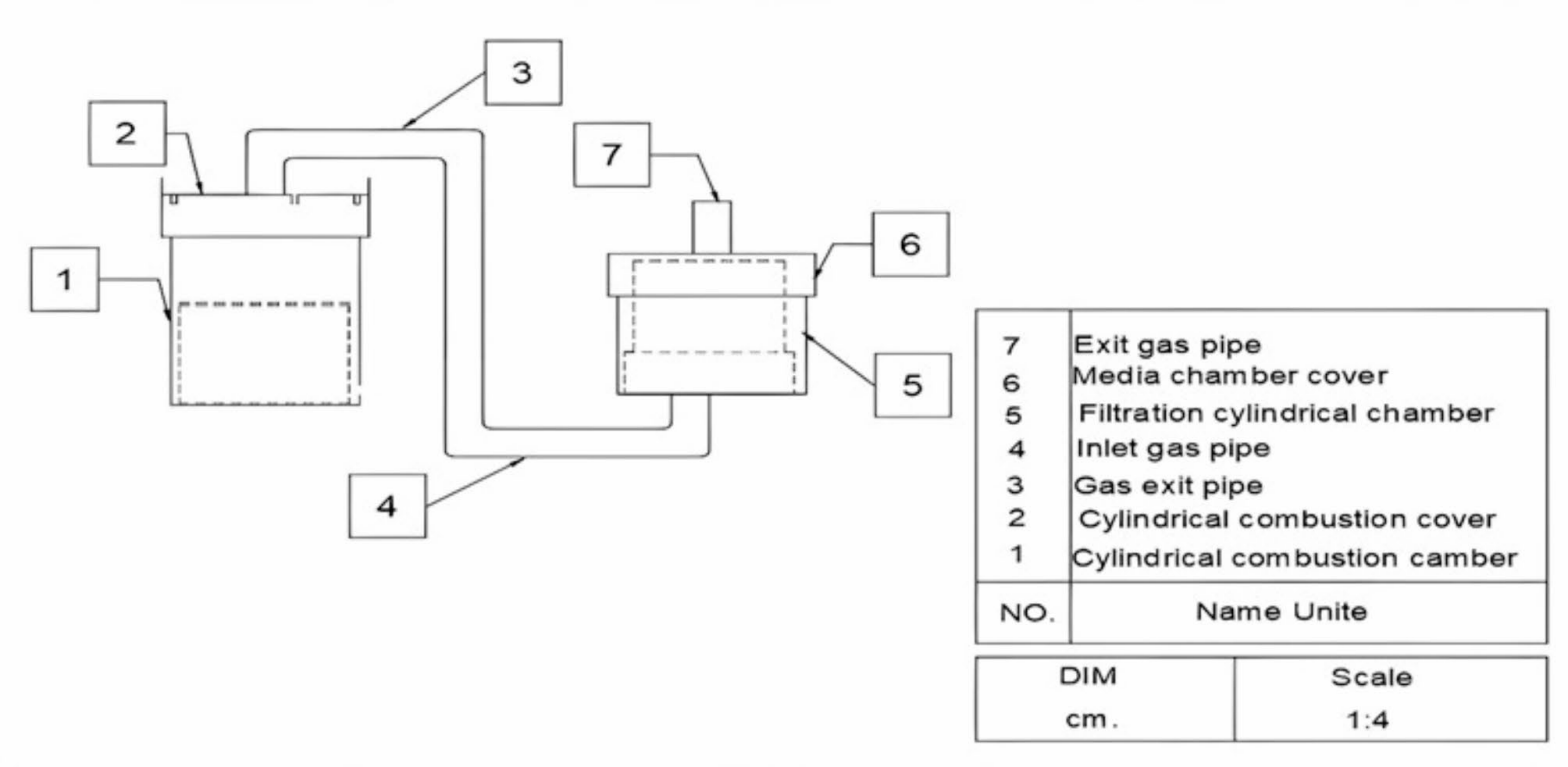
The test unit to simulate exhaust gas emissions.

This design, as illustrated in Fig. 3, simulates the exhaust gas filtration process, ensuring comprehensive testing of the proposed materials.

The adsorption efficiency can be computed by comparing the initial gas emission with the final gas concentration. The adsorption ratio can be calculated using Eq. (1)^16^.

$$\:\%Adsorption=\frac{{G}_{O}-{G}_{n}}{{\text{G}}_{\text{O}}}\times\:100\text{\%}\:\:\:\:\:\:\:\:\:\:\:\:\:\:\:\:\:\left(1\right)$$

The experiment was conducted at the Agricultural Engineering Research Institute, Research Center, Ministry of Agriculture. Initially, the gases emitted from burning activated charcoal mixed with diesel fuel were measured. Emissions, including (CO), (CO₂), and (HC), were analyzed both before and after the installation of the various media. Measurements were taken using an exhaust analyzer (AUTOCHEK-974/5) (SPTC) from the institute’s instrumentation laboratory.

### Characterization tests

#### Analysis of AC and AC + MgO

The concentrations of nitrates, sulfates, and carbonates in the samples (AC) and (AC-MgO) were quantified both before and after the treatment process. The analysis was conducted using a Kjeldahl apparatus (BOCHI-320, China) and a spectrophotometer (V730, China) at the National Research Center.

#### SEM and EDAX characterization of Ni-CNTs

Field emission scanning electron microscopy (FE-SEM, QUANTA EG) was used to examine the surface morphology and perform elemental analysis of Ni-CNTs nanocomposite coatings at varying concentrations. Energy-dispersive X-ray spectroscopy (EDX) was utilized to quantify the CNTs concentration as a percentage of the total coating weight and assess the adsorption efficiency of the Ni-CNTs nanocomposite coating.

#### RCBD analysis for treatment evaluation

A randomized complete block design (RCBD) with four factors and three replications for each parameter was used to analyze the data. Treatment means were compared using the least significant difference (L.S.D.) test, following established methodologies. Statistical analysis was performed using the Assistant software program to ensure accuracy and reliability in evaluating the effects^27^.

## Results & discussions

### Coating thickness and electroless deposition of Ni-CNTs

**Fig. 4:**
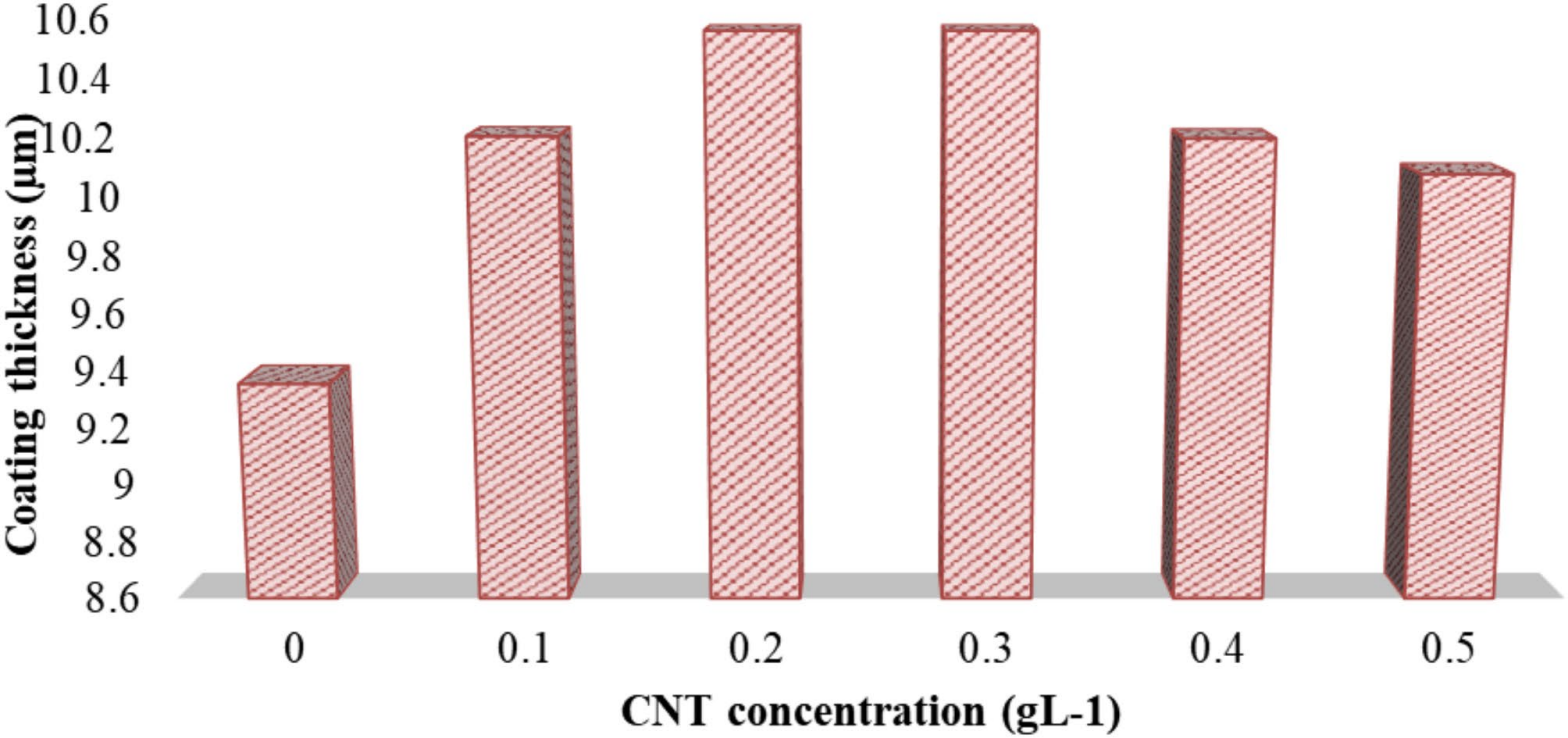
The coating thickness with different CNT concentrations.

The coating thickness during the electroless plating process was measured at different concentrations of carbon nanotubes (Ni-CNTs). As shown in Table 2; Fig. 4, the coating thickness slightly exceeded that of pure nickel as the concentration of Ni-CNTs increased, reaching up to 0.5 g L⁻¹. In other words, as the concentration of carbon nanotubes in the solution increased, the thickness of the applied coating also increased. A chemical plating process, free of electricity, was used to deposit a layer of nickel and carbon nanotubes (Ni-CNTs) onto the metallic surface. This process relies on a continuous chemical reaction rather than an electric current, with catalysts in the solution facilitating the even deposition of nickel and MWCNTs on the surface.

The results indicated that the coating thickness gradually increased with the rise in MWCNT concentration, while maintaining good conductivity properties due to the hydroxyl groups formed on the CNT surfaces following treatment with nitric acid (HNO₃)^28,29^. However, the coating thickness decreased as the concentration of CNTs exceeded 0.4 g L⁻¹, though it remained larger than the coating thickness of pure Ni, as depicted in Fig. 4 ^26^. This occurrence can be attributed to the clustering of carbon nanotubes in the solution at higher concentrations. Nonetheless, the coating thickness exhibited a positive correlation with the CNT concentration.

**Table 2:** Relationshipe between CNT concentrations and the coatingthickness of Ni-CNTs on the sheet.

CNT Conentration (gL-1)	0	0.1	0.2	0.3	0.4	0.5
Coating thickness (µm)	9.36	10.208	10.56	1056	10.2	10.08

Electroless nickel plating is a chemical deposition method that relies on a self-sustaining redox reaction between a nickel source, typically nickel salts like nickel sulfate, and a reducing agent such as sodium hypophosphite. This process takes place in an aqueous solution and does not require an external electrical current, distinguishing it from electroplating.

The primary reaction in electroless nickel plating involves the reduction of nickel ions (Ni²⁺) to metallic nickel (Ni) by the reducing agent, with the simultaneous oxidation of the reducing agent. For example, in the case of sodium hypophosphite, the simplified reaction can be represented as^30^:

2 $$\:{Ni}^{+2}+2{H}_{2}P{O}_{2}^{-}+2{H}_{2}O\to\:Ni+2{H}_{2}P{O}_{3}^{-}+2{H}^{+}$$

**Fig. 5:**
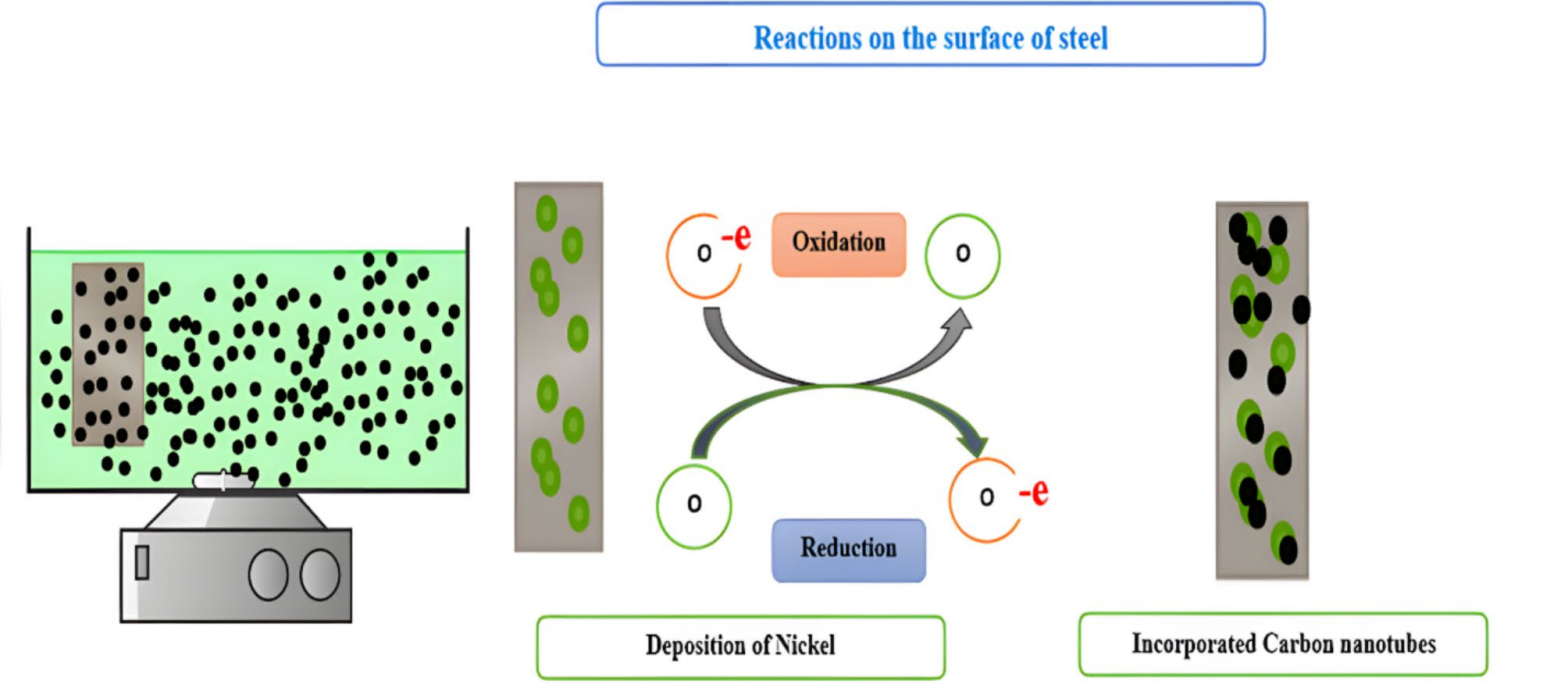
A diagram illustrating the fundamental reactions and processes involved in coating an acetylene plate with nickel and carbon nanotubes.

Whereas nickel ions ($\:{Ni}^{+2}$ ) are reduced to metallic nikel by hypophosphate ions ($\:2{H}_{2P}{O}_{2}^{-}$), which leads to the deposition of a nickel layer on the surface of stainless steel. The process of incorporating carbon nanotubes into the coating involves mixing them into the coating solution. Typically, this procedure involves modifying the surfaces of the carbon nanotubes to improve their dispersion and adherence to the metal surface. During the coating process, the carbon nanotubes are incorporated and firmly fixed within the nickel layer that is applied. The chemical linkages and interfacial interactions between the nickel and the modified surfaces of the CNTs play a crucial role in stabilizing the nanotubes within the coating. These interactions ensure that the CNTs are well-dispersed throughout the nickel matrix, contributing to the overall performance and durability of the coating^31^. Figure 5 presents a schematic representation of the fundamental reactions and processes involved in coating an acetylene plate with nickel and carbon nanotubes (CNTs). The diagram highlights the key stages of the electroless plating process, including the chemical reactions facilitating nickel deposition and the incorporation of CNTs into the nickel layer. It also illustrates the interactions between the modified CNT surfaces and the nickel matrix, ensuring uniform dispersion and enhanced adhesion. This visual aids in understanding the detailed mechanism of the coating process.

**Fig. 6:**
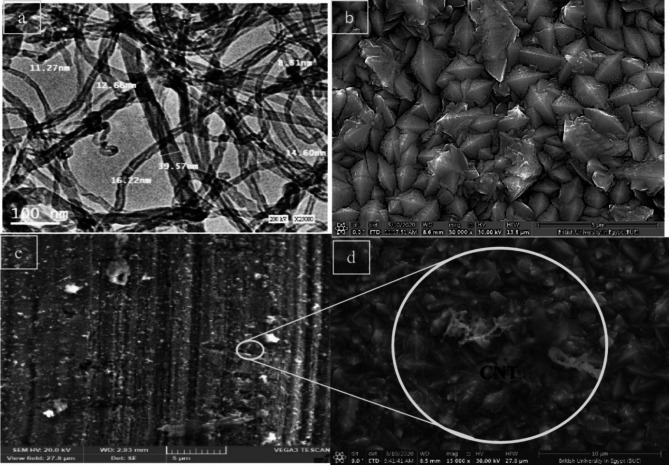
**(a**) TEM images of CNTs, (**b**) SEM images of Ni, (**c**) Ni-CNTs from bath containing 0.2 g.L^− 1^ CNTs, (**d**) CNTs on the coating surface.

The TEM analysis of the CNTs used in this study, shown in Fig. 6, revealed that the average diameter of the CNTs ranged from 10 to 40 nm, with lengths reaching up to 5 μm. Figure 6b–c illustrate the surface morphology of Ni-coated steel and Ni–CNTs nanocomposite-coated steel created through the electroless process at 90 °C and 250 rpm. The SEM image (Fig. 6b) demonstrated that the pure Ni coating exhibited a well-defined pyramidal structure, which represents the ideal morphology for Ni coatings. Additionally, it was observed that the morphology of Ni–CNT composite coatings with varying CNT concentrations (Fig. 6c–d) was finer compared to pure Ni coatings, attributed to the successful incorporation of CNTs into the Ni matrix. SEM micrographs revealed a uniform distribution of CNTs and a fully covered substrate, with no surface cracks or voids, especially at a CNT concentration of 0.4 g/L in the coating bath.

The influence of the concentration of CNTs in the coating bath on the weight% of CNTs in the nickel-coating matrix was examined using EDX. It was found that the presence of CNTs in the Ni matrix was significantly influenced by the concentration of CNTs in the coating solution. The weight% of CNTs in the coating layer exhibited a notable increase as the concentration of CNTs in the coating bath increased. The highest value, 15.63 wt%, was achieved at a concentration of 0.2 g L⁻¹. However, further increases in CNT concentration led to a decrease in the weight% of CNTs in the coating layer. The incorporation of CNTs into the Ni matrix is dependent on the quantity of CNTs present in the coating bath. When the concentration of CNTs in the electrolyte exceeds 0.4 g L⁻¹, clumps of CNTs form in the solution, impeding their integration into the desired material. These clumps are challenging to incorporate and hinder the dispersion of CNTs on the surface.

### Ni Catalysis in exhaust gas reactions

The Ni acts as a catalyst for several reactions involving common exhaust gases such as CO, NOx, and HC^32^.

- Oxidation of CO :

Ni can catalyze the oxidation of CO to CO₂.

3 $$\:2CO+{O}_{2}\to\:2{\text{C}\text{O}}_{2}$$

- Reduction of NOx:

Ni can also facilitate the reduction of NOx to N₂ and O₂ or to nitrogen and water in the presence of a reducing agent such as H₂.

4 $$\:2NO+2CO\to\:{N}_{2}+2C{O}_{2}$$

5 $$\:NO+CO\to\:\frac{1}{2}{N}_{2}+C{O}_{2}$$

- Oxidation of HCs:

Ni can help oxidize HC into CO_2_ and H_2_O.

6 $$\:{C}_{x}{H}_{y}+(x+\frac{y}{4})O2\to\:xCO2+\frac{y}{2}{H}_{2}O$$

### Role of CNTs in physical adsorption and catalytic enhancement

CNTs possess a high surface area and can effectively adsorb various gases^33^.

- Physical Adsorption.

CNTs can physically adsorb exhaust gases such as CO₂, CO, NOx, and VOCs due to their extensive surface area and porosity.

- Enhancement of Catalytic Activity.

The combination of CNTs and Ni enhances catalytic and adsorption properties. CNTs The incorporation of CNTs increases the number of active catalytic sites, thereby enhancing the efficiency of oxidation and reduction reactions. Moreover, CNTs offer structural reinforcement and thermal stability to Ni particles, ensuring reliable performance under the high-temperature conditions typical of exhaust systems. This synergistic interaction results in more efficient catalytic processes, as demonstrated in earlier studies^34^.

### Measurements and evaluation of the different media

#### Test unit results

The experiment was conducted at the Agricultural Engineering Research Institute, Research Center, Ministry of Agriculture, Egypt. Initially, gases emitted from burning activated charcoal mixed with diesel fuel were measured both before and after introducing various media. During this process, the following gases were assessed using different processing materials:

**a) Effect of Treatment Materials on CO**_**2**_ **Reduction**.

**Fig. 7:**
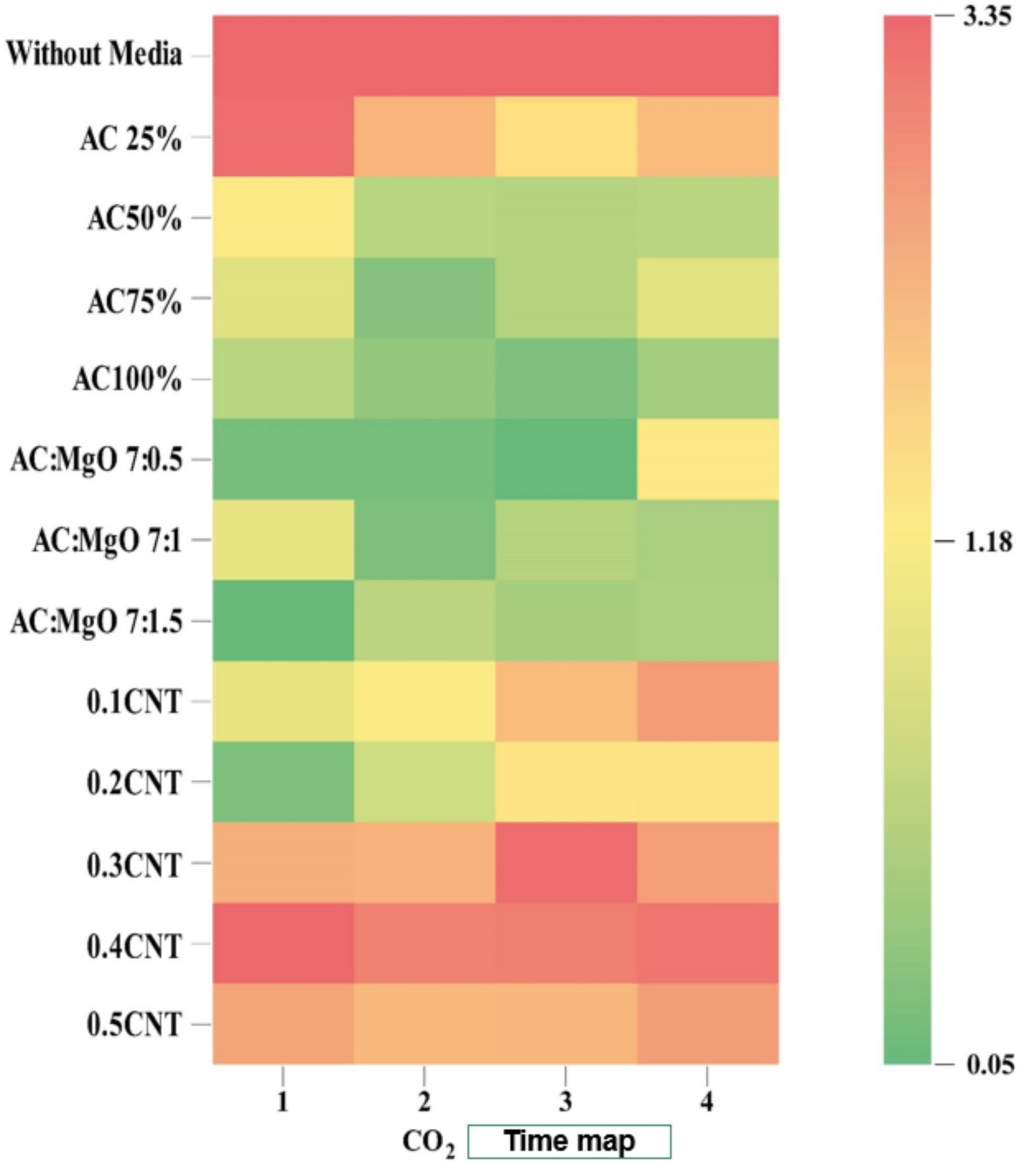
The impact of various treatment materials on the levels of$\:\:C{O}_{2}$.

When measuring carbon dioxide gas using treatment materials in various proportions, the results were as shown in Fig. 7, which illustrates the impact of different treatment materials on CO_2_ levels.

The results indicate that increasing the concentration of activated carbon (AC) enhances the adsorption efficiency for CO₂, with efficiencies of 78.34% at 25% concentration and 85.21% at 100%. Furthermore, the addition of magnesium oxide (MgO) to the AC improved adsorption, with the optimal ratio of 7:0.5 AC to MgO achieving an adsorption efficiency of 86.84% for CO₂. This suggests that MgO contributes to the overall efficiency, possibly by enhancing the surface properties of AC, as reported in previous studies on composite adsorbents^35^. Additionally, the highest adsorption efficiencies for CO₂ were observed with Ni-CNTs at a concentration of 0.2, achieving 93.13%, which is consistent with findings from studies on carbon nanotube composites, where the high surface area and reactivity of Ni-CNTs significantly improve adsorption performance ^36^. These results underscore the synergistic effect of using hybrid materials such as MgO and Ni-CNTs to enhance adsorption efficiencies for CO₂ capture.

**b) Effect of treatment materials on CO reduction**.

**Fig. 8:**
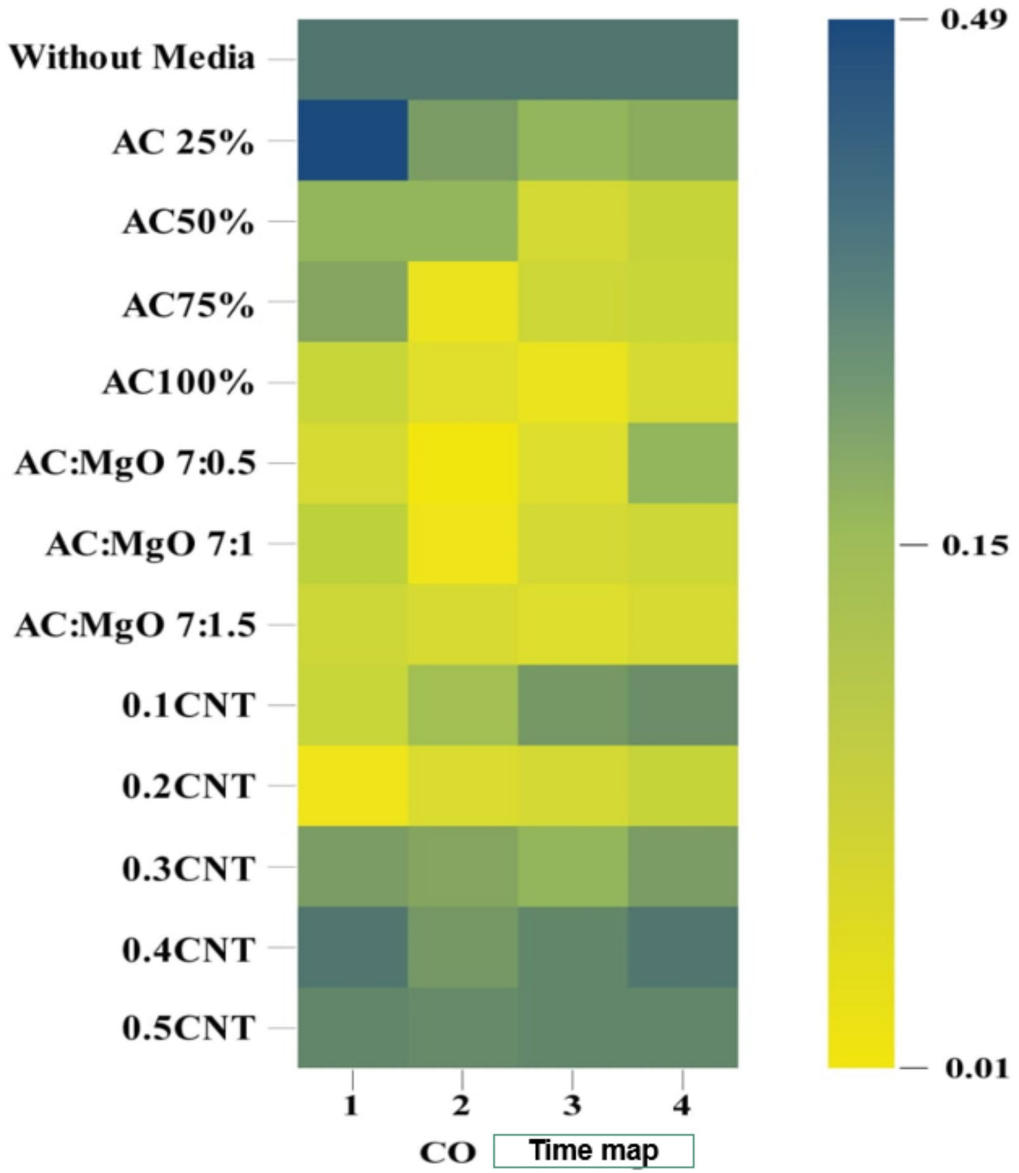
The impact of various treatment materials on the levels of$\:\:CO$.

When measuring CO gas using treatment materials in various proportions, the results were as shown in Fig. 8, which illustratesthe impact of different treatment materials on CO levels.

The results showed that at 100% activated carbon (AC) concentration, the CO adsorption efficiency was 80.77%. When magnesium oxide (MgO) was introduced, the adsorption efficiency was enhanced compared to AC alone, with the optimal ratio of 7:0.5 AC to MgO achieving 76.92% for CO. Additionally, the Ni-CNT_S_ Coating demonstrated the highest adsorption efficiency of 94.87% for CO at a concentration of 0.2, highlighting the significant impact of nanomaterial integration on adsorption performance. This suggests that the integration of nanomaterials like Ni-CNTs significantly boosts adsorption performance, aligning with studies that show the synergistic effect of combining these materials to improve adsorption properties^37^.

**c) Effect of treatment materials on HC reduction**.

**Fig. 9:**
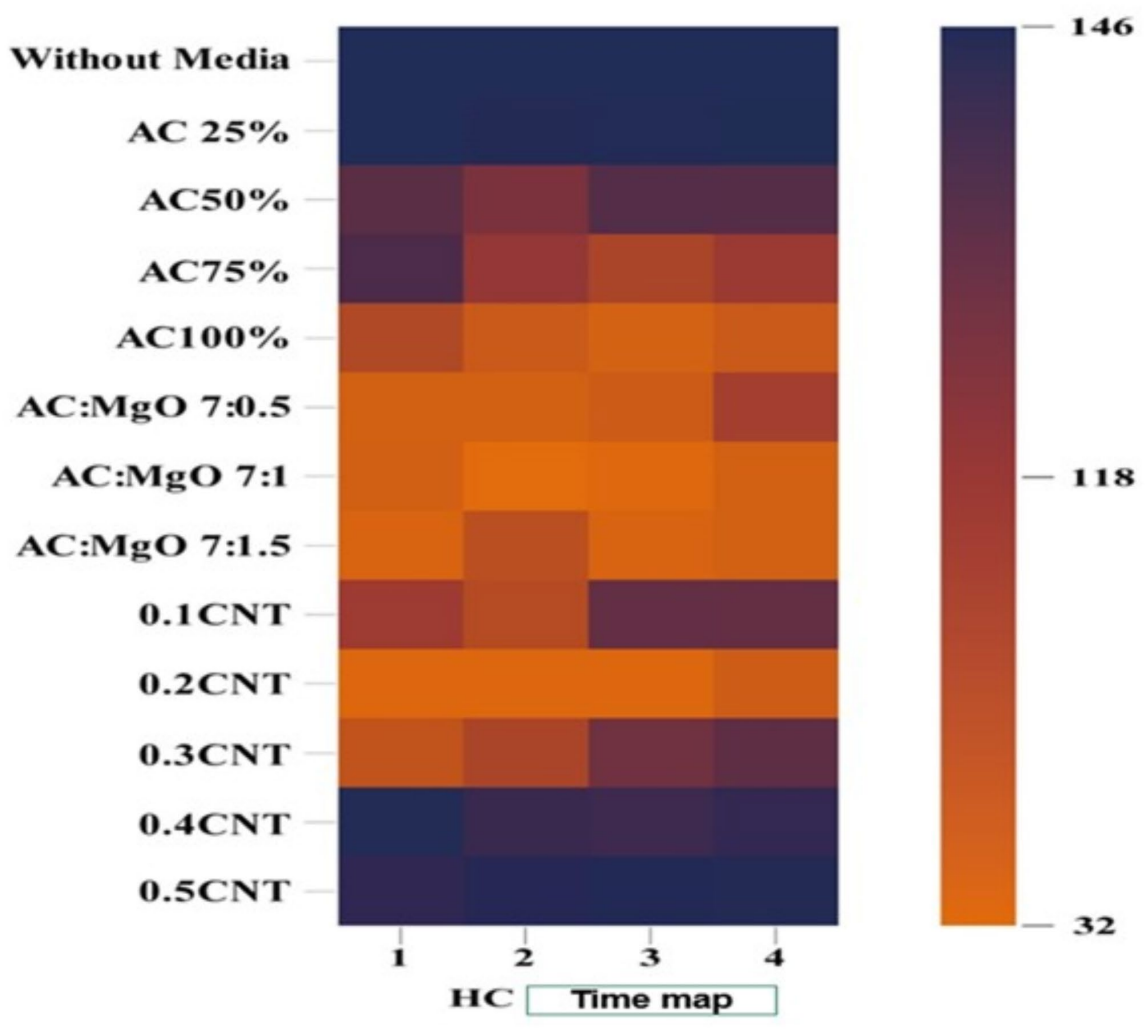
The impact of various treatment materials on the levels of$\:\:HC$.

When measuring $\:HC$ using treatment materials in various proportions, the results were as shown in Fig. 9, which illustrates the impact of different treatment materials on $\:HC\:$levels

The results revealed that at a 100% concentration of (AC), the adsorption efficiency for (HC) was 68.84%. The use of a blend of AC and MgO) at various ratios showed improved adsorption performance compared to using AC alone. The optimal ratio of 7:1.5 (AC: MgO) achieved an efficiency of 74.65% for HC. Additionally, the highest adsorption efficiency of 76.02% for HC was achieved using Ni-CNTs at a concentration of 0.2, underscoring their superior performance in HC treatment.These gases were evaluated to understand their changes and the impact of different media on emissions. This structured approach ensures the reliability of data collected for further analysis and research.

#### Chemical characterization of AC and AC + MgO

The chemical analysis of (AC) and (AC + MgO) samples, conducted using the Kjeldahl BOCHI-320 and Spectrophotometer V730, aimed to determine the concentration of nitrates (NO₃), sulfates (SO₄), and carbonates (CO₃) before and after the filtration treatment. These analyses, performed at the Central Laboratory Network (CLN) of the National Research Center, serve to evaluate the efficiency of NO_x_ and SO_2_ adsorption by these materials.

**Fig. 10:**
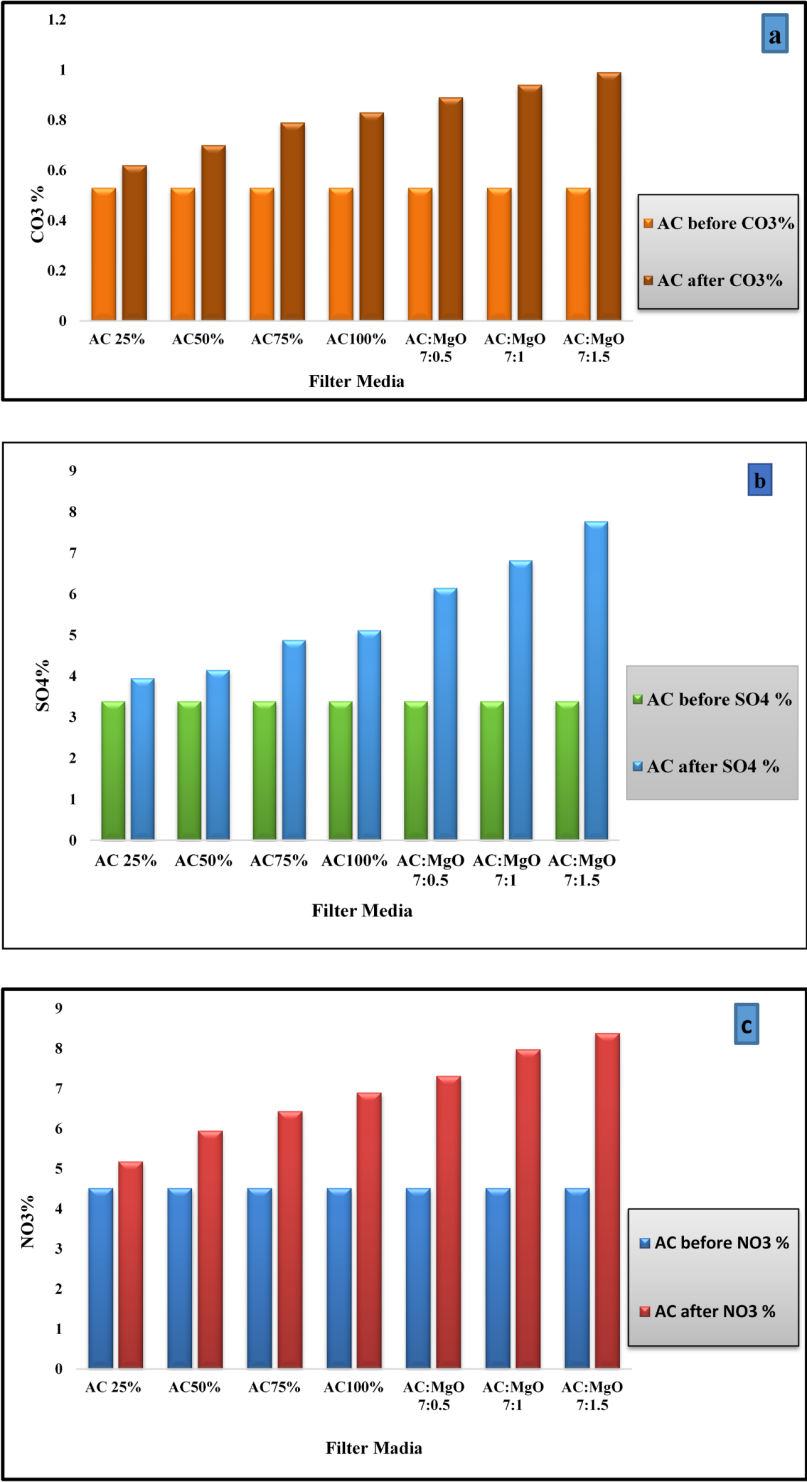
(**a**) The comparison of ($\:C{O}_{3}\%)$& (**b**$\:\left)\:\:\right(S{O}_{4}\%)\:$and (**c**) $\:(N{O}_{3}\%$) in ($\:AC\:\&\:AC+MgO)$ before and after Filtratio.

By assessing the percentage of NO₃, SO₄ and CO₃ in the filtrate samples before and after filtration, the experiment provides insight into the extent to which these pollutants were adsorbed. The comparison of nitrate, sulfate, and carbonate concentrations in both the AC and AC + MgO media is visually depicted in Fig. 10. This comparison allows for a clear understanding of how effectively the different filtration mediums adsorb harmful gases such as nitrogen oxides (NO_x_) and sulfur dioxide (SO₂). Such detailed chemical analyses help in confirming the contribution of each component (AC and MgO) to the overall efficiency of the filtration system in reducing air pollution. By monitoring the changes in the composition of these ions, the effectiveness of the treatment process is quantitatively assessed.

The results of the chemical analysis revealed that the higher concentration of MgO in the activated carbon medium significantly enhanced its efficiency in converting harmful gases, such as (NO_x_), (SO₂), and (CO and CO₂), into their corresponding salts—nitrates (NO₃⁻), sulfates (SO₄²⁻), and carbonates (CO₃²⁻), respectively. This transformation is attributed to the catalytic activity of MgO, which facilitates the chemical reactions responsible for gas adsorption and subsequent salt formation. The data indicate that increasing the MgO content not only improved the medium’s adsorption capacity but also contributed to a more efficient conversion process, effectively reducing the concentrations of toxic gases in the treated emissions^38^.

**Fig. 11:**
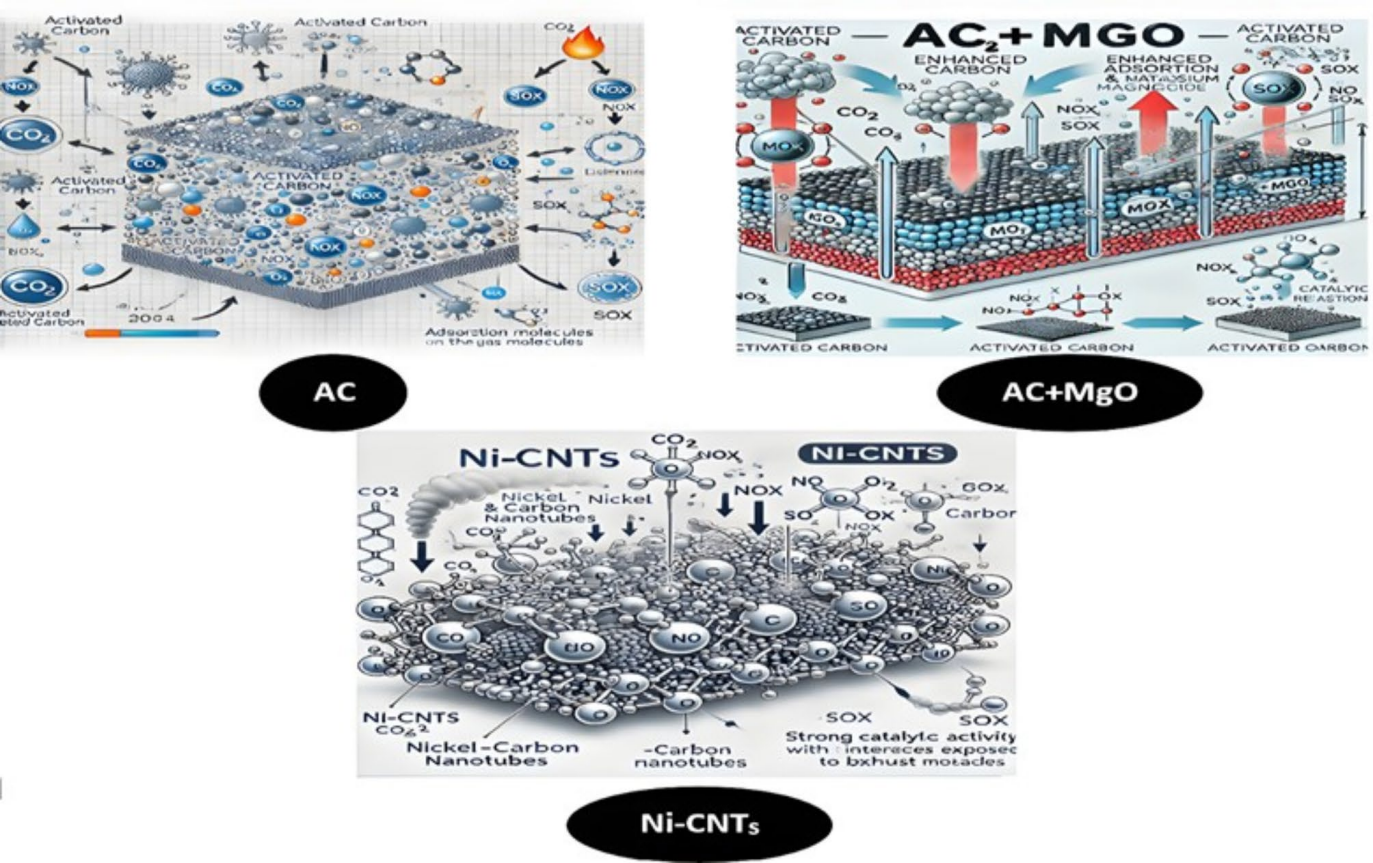
The reactions occurring on the surface of$\:AC$, $\:AC+MgO$, and (Ni-CNTs)surfaces when exposed to various gases Generated using ChatGPT (2024).

Figure 11 illustrates the reactions occurring on the surface of activated carbon, activated carbon combined with magnesium oxides, and (Ni-CNTs) surfaces when exposed to various gases.

Activated carbon, when utilized for adsorbing toxic gases or harmful exhaust emissions, facilitates two primary interaction mechanisms on its surface:

- Physical Adsorption: Molecules adhere to the activated carbon surface through weak intermolecular forces, such as Van der Waals forces. This mechanism effectively captures small gas molecules, including (O₂), (N₂), and (CO₂), by creating a large surface area and enabling reversible adsorption without altering the chemical structure of the gases^39^.
- Chemisorption: This type of adsorption includes chemical reactions between gaseous molecules and active sites on the activated carbon surface. These reactions can lead to the formation of new chemical bonds, for example, reacting with NO_X_, SO_X_, or other organic pollutants.

Physical adsorption and chemisorption are crucial mechanisms by which activated carbon effectively captures and removes harmful gases from the environment^40^.

Potential chemical reactions between ($\:MgO$) and ($\:CO$), $\:\left(C{O}_{2}\right)$, $\:\left(N{O}_{X}\right)$, and $\:\left(S{O}_{2}\right)$ are provided below:

- ($$\:CO$$):

7 $$\:MgO+C0\:\to\:Mg+C{O}_{2}$$

b) ($$\:C{O}_{2}$$):

8 $$\:MgO+C{O}_{2}\to\:MgC{O}_{3}$$

c) ($$\:NOx$$):

Reactions with$\:\:NOx$, in the presence of$\:\:MgO$, can be complex, involving reduction to convert $\:NOx$ to $\:{N}_{2}$ and$\:{O}_{2}$, or oxidation to form nitrogen-containing compounds like $\:N{O}_{2}$ or$\:N{O}_{3}$.

9 $$\:2MgO+N{O}_{X}\to\:M{g}_{2}O+{N}_{2}$$

10 $$\:MgO+N{O}_{X}\:\to\:MgN{O}_{3}$$

d) ($$\:S{O}_{2}$$)

11 $$\:MgO+S{O}_{2}\to\:MgS{O}_{3}\:or\:MgS{O}_{4}$$

Overall, impregnating activated carbon with magnesium oxide enhances its adsorptive capabilities, making it more effective in mitigating environmental pollutants from exhaust gases^41^.

A sheet of paint composed of Ni-CNT_S_, designed to adsorb exhaust gases, will facilitate several reactions due to the catalytic properties of nickel and the high surface area and unique characteristics of CNTs. The combination of these two materials creates an effective system for gas absorption and conversion. Ni as a catalyst, plays a significant role in accelerating reactions with toxic exhaust gases such as (NOx), (CO), and (HC). Ni can promote the reduction of NOx into less harmful compounds, while its catalytic activity also helps break down CO and HC molecules^42^.

#### SEM and EDAX analysis of Ni-CNTS nanocomposite coating

**Fig. 12:**
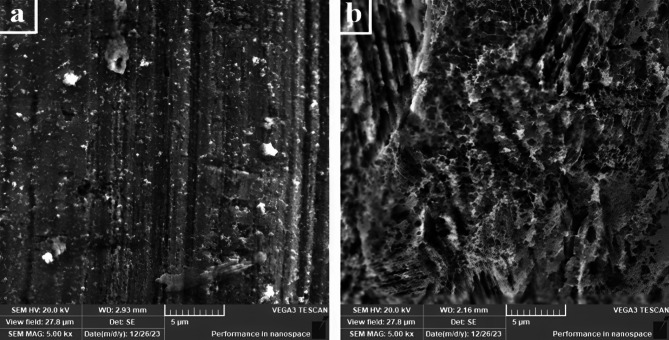
SEM for a concentration of 0.1(Ni-CNT_S_) (**a**) before and (**b**) after filtration.

**Fig. 13:**
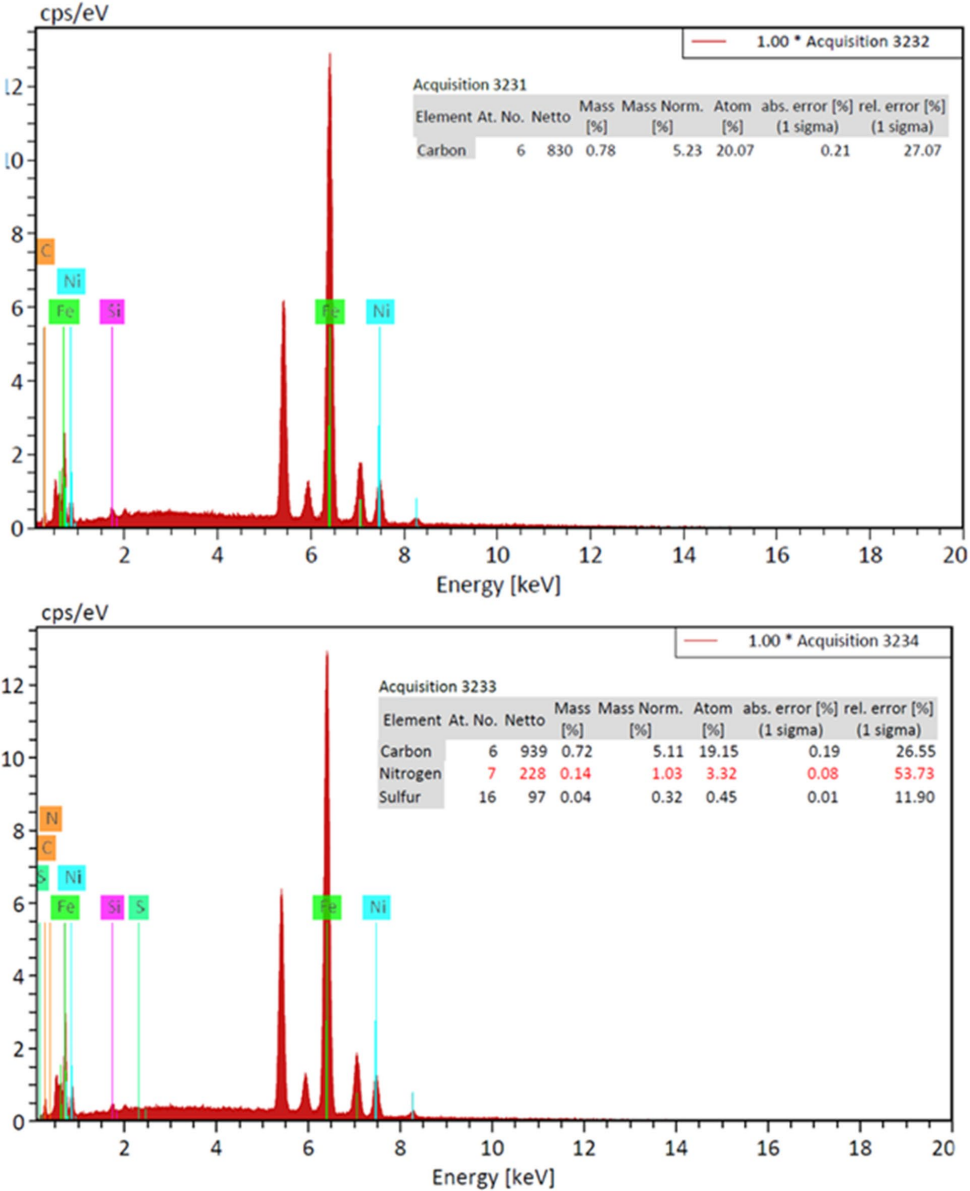
EDEX for a concentration of 0.1(Ni-CNT_S_) before and after filtration.

**Fig. 14:**
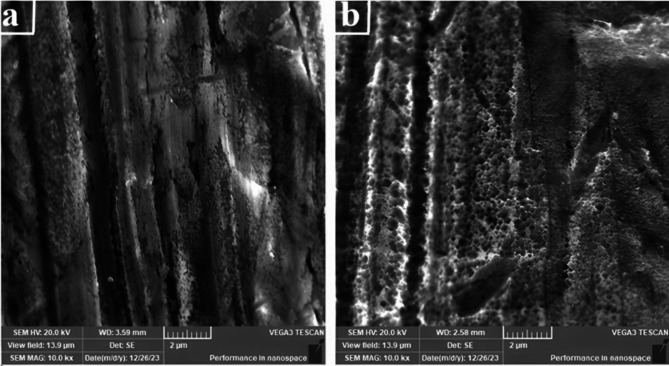
SEM for a concentration of 0.2 (Ni-CNT_S_) (**a**) before and (**b**) after filtration.

**Fig. 15:**
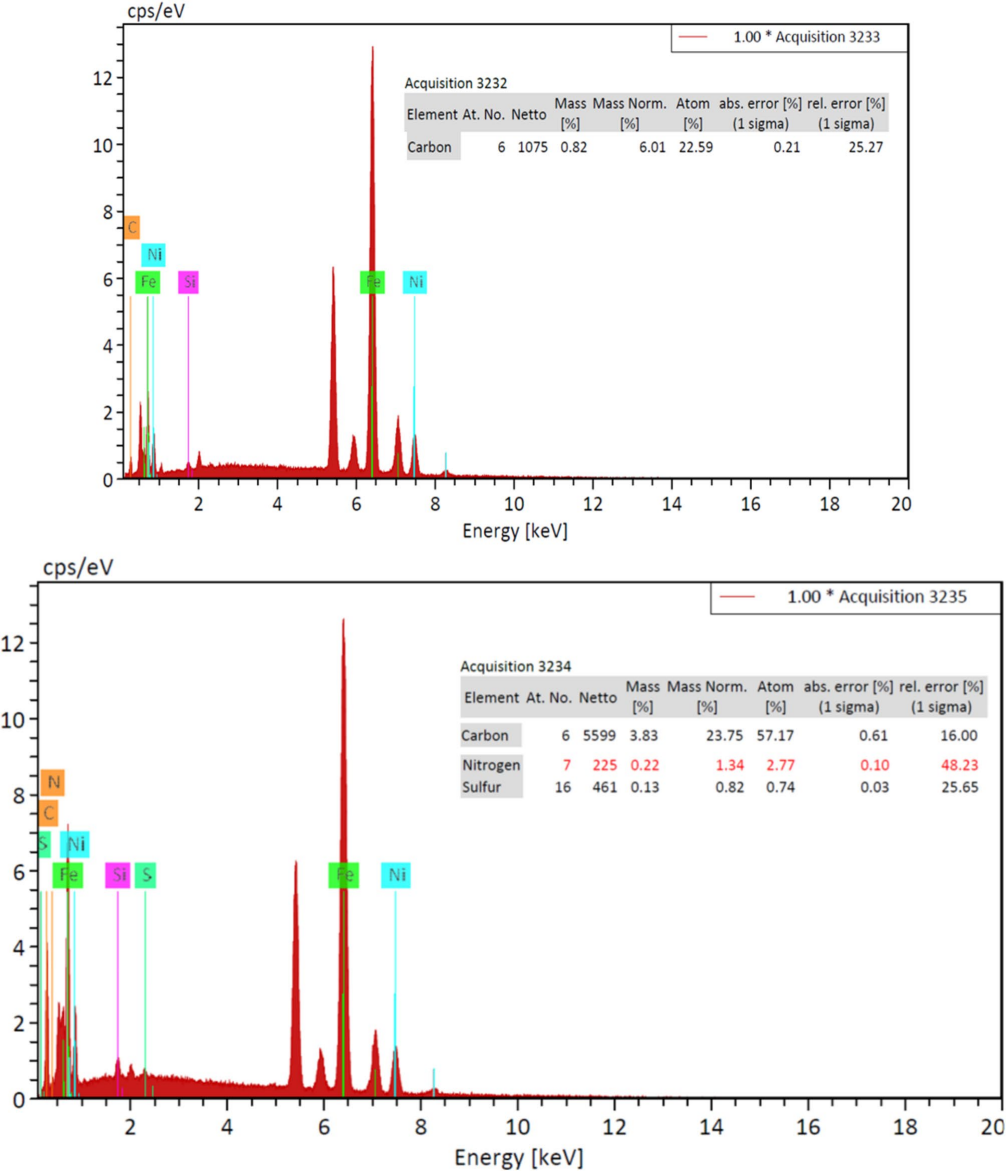
EDEX for a concentration of 0.2(Ni-CNT_S_) before and after filtration.

SEM and EDAX techniques were utilized to evaluate the adsorption process on the nanosheets. The surface morphology of the nanosheets before and after adsorption was examined using a scanning electron microscope (TESCAN VEGA3) at the National Research Center - Center of Excellence for Medical Research. Electronic scans were performed on the most effective nanosheets for adsorption, specifically those coated with 0.1 and 0.2 Ni-Carbon Nanotube Nanocomposite. Figures 12 and 13 illustrate the results for the nanosheet coated with 0.1 Ni-Carbon Nanotube Nanocomposite before and after the filtration process. Similarly, Figs. 14 and 15 present the findings for the nanosheet coated with 0.2 Ni-Carbon Nanotube Nanocomposite before and after filtration, as presented in Table 3. Specific observations include:

- SEM analysis at a concentration of 0.1 Ni-CNT_S_ Nanocomposite Coating revealed noticeable changes in the surface morphology of the acetylene sheet before and after gas adsorption. These changes indicate the successful interaction between the nanosheet surface and the adsorbed gases. Furthermore, EDX analysis demonstrated the presence of N% (1.03%) and S% (0.32%) elements after the filtration process, providing strong evidence for the adsorption of (NOx) and (SO2). The detection of these elements supports the hypothesis that the nanocomposite coating effectively captures these pollutants during the filtration process.
- SEM analysis at a concentration of 0.2 Ni-CNT_S_ Nanocomposite Coating revealed significant changes in the surface texture of the acetylene sheet before and after gas adsorption, indicating successful interaction between the surface and the adsorbed gases. EDX analysis showed the presence of N% (1.34%) and S% (0.82%) after the filtration process, suggesting the adsorption of NOx and SO2 at concentrations higher than those observed with 0.1 Ni-CNT_S_. This highlights the enhanced adsorption capability at the 0.2 Ni-CNT_S_ concentration. Additionally, there was a marked increase in the carbon content, reflecting the adsorption of CO, CO_2_ and HC. The carbon content increased from 6.01 to 23.75%, further emphasizing the improved efficiency of adsorption at this higher concentration.

**Table 3:** Shows the concentration of chemical elements before and after the filtration process.

Element	Before (0.1) Ni-CNTS	After (0.1) Ni-CNTS	Before (0.2) Ni-CNTS	After (0.2) Ni-CNTS
C	**5.23**	**5.11**	**6.01**	**23.75**
N	-	**1.03**	-	**1.34**
S	-	**0.32**	-	**0.82**

#### Statistical analysis of filtration media performance

The tables (Tables 4, 5, 6 and 7) present the mean performance data for various filtration media in reducing diesel and gas emissions over different time intervals.

**Table 4:** Mean performance of filtration media diesel emission and G gasemission for filtration.

Filtration media	Gas emission			Mean
	Co2	Co	Hc	
Without Media	3.35 h	0.39 h	146.00 a	49.91 a
AC 25%	3.28 h	0.49 h	146.00 a	49.92 a
AC50%	1.26 h	0.24 h	99.00 c	33.50 c
AC75%	1.02 h	0.28 h	107.00 c	36.10 bc
AC100%	0.73 h	0.12 h	56.00 de	18.95 de
AC: MgO 7:0.5	0.20 h	0.09 h	39.00 fg	13.10 e
AC: MgO 7:1	1.09 h	0.15 h	40.00 fg	13.75 e
AC: MgO 7:1.5	0.07 h	0.11 h	37.00 fg	12.39 e
0.1 Ni-CNTs	1.10 h	0.12 h	64.00 d	21.74 d
0.2 Ni-CNTs	0.23 h	0.03 h	35.00 g	11.75 e
0.3 Ni-CNTs	2.23 h	0.30 h	48.00 ef	16.84 de
0.4 Ni-CNTs	3.35 h	0.39 h	143.00 a	48.91 a
0.5 Ni-CNTs	2.37 h	0.35 h	121.00 b	41.24 b
**Mean**	1.56 b	0.24 b	83.15 a	

LSD value at 0.05 Filtration media: 7.3581 Gas emission: 3.5347 Interaction: 12.745.

The table presents the results of the statistical analysis for filtration media and gas emissions, with a control group (without media) over a period of 1 min. The analysis revealed significant effects across all filtration media, with the most effective being AC: MgO at all concentrations and Ni-Carbon Nanotube Nanocomposite at 0.2% CNT.

**Table 5:** Mean performance of filtration media diesel emission and gas emission for filtration.

Filtration media	Gas emission			Mean
	Co2	Co	Hc	
Without Media	3.35 j	0.39 j	146.00 a	49.91a
AC 25%	2.23 j	0.30 j	135.00 ab	45.84 ab
AC50%	0.73 j	0.24 j	82.00 d	27.66 c
AC75%	0.31 j	0.03 j	69.00 e	23.11 cd
AC100%	0.44 j	0.06 j	44.00 g-i	14.83 efg
AC: MgO 7:0.5	0.20 j	0.01 j	39.00 hi	13.07 efg
AC: MgO 7:1	0.24 j	0.02 j	32.00 i	10.75 g
AC: MgO 7:1.5	0.75 j	0.09 j	51.00 f-h	17.28 d-g
0.1CNTs	1.28 j	0.21 j	54.00 fg	18.50 def
0.2CNTs	0.89 j	0.08 j	35.00 i	11.99 fg
0.3CNTs	2.18 j	0.28 j	58.00 ef	20.15 de
0.4CNTs	2.92 j	0.31 j	114.00 c	39.08 b
0.5CNTs	2.07 j	0.34 j	125.00 bc	42.47 b
**Mean**	1.35 b	0.18 b	75.69 a	

LSD value at 0.05 Filtration media: 7.2285 Gas emission: 3.4724 Interaction: 12.520.

The table presents the results of the statistical analysis for filtration media and gas emissions, with a control group (without media) over a period of 2 min. The analysis revealed significant effects across all filtration media, with the most effective being AC: MgO at 7:1 concentrations and Ni-Carbon Nanotube Nanocomposite at 0.2 Ni-CNTs.

**Table 6:** Mean performance of filtration media diesel emission and gas emission for filtration.

Filtration media	Gas emission			Mean
	Co2	Co	Hc	
Without Media	3.34 g	0.39 g	146.00 a	49.91 a
AC 25%	1.50 g	8.49 g	140.00 ab	50.00 a
AC50%	0.70 g	0.10 g	102.00 cd	34.27 cd
AC75%	0.71 g	0.11 g	58.00 e	19.61e
AC100%	0.26 g	0.03 g	38.00 f	12.76 e
AC: MgO 7:0.5	0.05 g	0.07 g	43.00 f	14.37 e
AC: MgO 7:1	0.70 g	0.10 g	34.00 f	11.60 e
AC: MgO 7:1.5	0.59 g	0.07 g	37.00 f	12.55 e
0.1 Ni-CNTs	2.01 g	0.31 g	94.00 d	32.11 cd
0.2 Ni-CNTs	1.39 g	0.10 g	35.00 f	12.16 e
0.3 Ni-CNTs	3.26 g	0.24 g	88.00 d	30.50 d
0.4 Ni-CNTs	2.96 g	0.35 g	114.00 c	39.10 bc
0.5 Ni-CNTs	2.10 g	0.35 g	131.00 b	44.48 ab
**Mean**	1.51 b	0.82 b	81.54 a	

LSD value at 0.05 Filtration media: 8.1250 Gas emission: 3.9031 Interaction: 14.073.

The table presents the results of the statistical analysis for filtration media and gas emissions, with a control group (without media) over a period of 3 min. The analysis revealed significant effects across all filtration media, with the most effective being AC: MgO at all concentrations and Ni-Carbon Nanotube Nanocomposite at 0.2 Ni-CNTs.

**Table 7:** Mean performance of filtration media diesel emission and gas emission for filtration.

Filtration media	Gas emission			Mean
	Co2	Co	Hc	
Without Media	3.35 g	0.39 g	146.00 a	49.91 a
AC 25%	2.02 g	0.26 g	144.00 ab	48.76 a
AC50%	0.74 g	0.13 g	101.00 d	33.96 c
AC75%	1.04 g	0.12 g	65.00 e	22.05 d
AC100%	0.57 g	0.09 g	44.00 f	14.89 ef
AC: Mg O 7:0.5	1.31 g	0.24 g	62.00 e	21.18 de
AC: MgO 7:1	0.60 g	0.11 g	39.00 f	13.24 f
AC: MgO 7:1.5	0.63 g	0.09 g	39.00 f	13.24 f
0.1Ni-CNTs	2.53 g	0.33 g	93.00 d	31.95 c
0.2 Ni-CNTs	1.40 g	0.13 g	42.00 f	14.51 ef
0.3 Ni-CNTs	2.46 g	0.30 g	97.00 d	33.26 c
0.4 Ni-CNTs	3.13 g	0.39 g	120.00 c	41.17 b
0.5 Ni-CNTs	2.50 g	0.36 g	133.00 b	45.29 ab
**Mean**	1.71 b	0.23 b	86.54 a	

LSD value at 0.05 Filtration media: 6.6760 Gas emission: 3.2070 Interaction: 11.56.

The table presents the results of the statistical analysis for filtration media and gas emissions, with a control group (without media) over a period of 4 min. The analysis revealed significant effects across all filtration media, with the most effective being AC: MgO at (7:1&7:1,5) concentrations and Ni-Carbon Nanotube Nanocomposite at 0.2 Ni-CNTs.

The results presented in Tables 4, 5, 6 and 7 highlight the effectiveness of different filtration media in reducing gas emissions, with significant differences observed between the control group and the treatments using filtration media. These findings are robustly supported by statistical analysis, which identified notable and statistically significant variations in emission reductions between the different filtration media at specific concentrations.

For example, the combination of (AC-MgO) consistently demonstrated strong performance at all tested concentrations. This consistent behavior confirms that AC-MgO has a high capacity for adsorbing gaseous pollutants, making it an effective filtration medium. Similarly, Ni-CNTs at a concentration of 0.2 demonstrated substantial efficacy, positioning it as a promising option for reducing emissions.

To assess the statistical significance of the differences between treatments, the Least Significant Difference (L.S.D) test was employed. This test confirmed that the differences observed were not due to random chance but were indeed reflective of the true effectiveness of each filtration medium. The results indicate that various filtration media exhibit different levels of efficacy, with some proving more effective in reducing emissions than others.

The results, along with the statistical analysis, support the idea that both activated carbon with magnesium oxide and nickel-carbon nanotube composites are highly effective in reducing gas emissions. It has been shown that certain concentrations are optimal for achieving maximum filtration performance. This enhances the current knowledge regarding the role of advanced materials in exhaust gas treatment and provides a foundation for future research aimed at improving filtration technologies for environmental protection.

## Conclusion

From the final results of this main points were resulting many concluded as:-.

1. Activated carbon is the best material for absorbing emissions.
2. For optimal performance, the space containing the activated carbon must be completely filled.
3. Activated carbon combined with metal oxides, such as magnesium oxide, is more efficient in adsorption than activated carbon alone. A lower percentage of magnesium oxide mixed with activated carbon is more effective at adsorbing carbon monoxide and hydrocarbons, while higher percentages improve adsorption of nitrogen oxides and sulfur dioxide.
4. A concentration of 0.2 (Ni-CNTs) is the most efficient for absorbing the resulting emissions and capturing most gases.

## Future outlook

Future studies will focus on exploring the integration of advanced nanomaterials to further improve the efficiency of exhaust gas treatment. By tailoring nanomaterials with specific properties such as high surface area, targeted adsorption capabilities, and enhanced catalytic activity, significant advancements in the filtration process are anticipated. These future investigations will seek to identify and assess new nanomaterial combinations, potentially driving innovation and pushing the boundaries of exhaust purification technology. This will contribute to the development of more sustainable and effective solutions for controlling diesel engine emissions, ultimately advancing environmental protection and energy efficiency.

These future investigations will seek to identify and assess new nanomaterial combinations, potentially driving innovation and pushing the boundaries of exhaust purification technology. This will contribute to the development of more sustainable and effective solutions for controlling diesel engine emissions, ultimately advancing environmental protection and energy efficiency.

## Data Availability

The data that support the findings of this study are available from the corresponding author.

## Abbreviations

PM: Particulate Matter
CO: Carbon Monoxide
CO_2_: Carbon Dioxide
HC: Hydrocarbons
SO_2_: Sulfur Dioxide
NOx: Nitrogen Oxides
VOCs: volatile organic compounds
DPF: diesel particulate filters
SCR: selective catalytic reduction
EGR: exhaust gas recirculation
DOC: Diesel Oxidation Catalysts
MgO: magnesium oxide
AC: activated carbon
CNTs: carbon nanotubes
Ni-CNTs: Nickel-carbon nanotube
S %: Sulfur Content
N %: Nitrogen Content
C %: Carbon Content
Pb: Lead
As: Arsenic
Cd: Cadmium
Hg: Mercury
SEM: Scanning Electron Microscope
EDX: Energy Dispersive X-ray Spectroscopy
TEM: Transmission Electron Microscopy
Wt%: The total work
Si%: silicon
Mn%: manganese
Fe%: iron
P%: phosphorus
